# Insights From Observations and Large‐Scale Field Experiments on Vole Population Cycles in Northern Europe: A 40‐Year Study of Predator–Prey Interactions

**DOI:** 10.1002/ece3.71419

**Published:** 2025-05-08

**Authors:** Erkki Korpimäki, Peter B. Banks, Tero Klemola

**Affiliations:** ^1^ Department of Biology University of Turku Turku Finland; ^2^ School of Life and Environmental Sciences University of Sydney Sydney New South Wales Australia

**Keywords:** density dependence, food and intrinsic factor hypotheses, mechanistic approach, numerical and functional responses, predation, vole cycles

## Abstract

The mechanisms driving 3–5‐year population cycles of voles involve delayed density‐dependent feedback on vole populations. The key drivers of this feedback include prolonged periods of food depletion or predation mortality over more than one phase of the cycle. We review observational and experimental data gathered between the 1970s and 2010s on vole population fluctuations and the responses of their avian and mammalian predators in west‐central Finland, focusing on studies that have investigated these drivers. Least weasels and stoats were the main predators of voles, causing 77% of all kills, while 22% were killed by avian predators. The numbers of least weasels tracked vole densities with a 9–12‐month lag, which resulted in delayed density‐dependent kill rates of voles in winter. Experimental reduction of small mustelids and avian predators in unfenced areas (each 2.5–3 km^2^) prevented the cyclic decline of vole densities in the subsequent summer, whereas in areas with only least weasel reduction and in control areas, a decline in vole densities occurred. In another field experiment, the reduction of both mustelid and avian predator densities increased the autumn density of *Microtus* voles fourfold during the low phase of the cycle, accelerated the increase twofold, increased the autumn density of voles twofold in the peak phase, and delayed the initiation of decline. Our unique experimental results suggest that the collective impact of both mustelid and avian predators is a likely mechanistic explanation for high‐amplitude population cycles of voles in North Europe. In these highly seasonal environments with short summers, a shortage of high‐quality winter food may be the directly density‐dependent factor stopping the growth of vole populations. This allows predators to catch up with prey densities and impose population decline and prolong the low phase of the cycle in a delayed density‐dependent manner.

## Graphical abstract

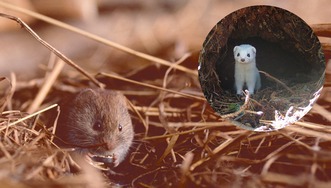

## 1 Introduction

Periodic population fluctuations (3–5‐year cycles) of small rodents (voles and lemmings) in boreal and arctic areas were first described > 170 years ago (Ehrström 1852; Collett 1878) and studied at least for 100 years (Elton 1924; Hansson and Henttonen 1988; Norrdahl 1995), but the exact mechanisms driving these population cycles have remained elusive (e.g., Krebs 2013; Myers 2018; Andreassen et al. 2021). It is, however, generally assumed that mechanisms generating cyclic population signatures include some sort of delayed density‐dependent feedback on rodent populations, such as prolonged periods of food depletion or predation mortality over more than one phase of the cycle (Stenseth 1999; Hanski et al. 2001; Myers 2018).

Because a delayed density‐dependent feedback factor must have an effect on the birth and/or death rate of rodents (e.g., Henttonen et al. 1987; Hanski et al. 1993; Korpimäki 1993; review in Klemola et al. 2003), trophic interactions with food supply or with natural enemies (predators, parasites, pathogens) are put forward as likely candidates driving these population cycles (e.g., Henttonen et al. 1987; Korpimäki 1993; Batzli 1996; Stenseth 1999; Korpimäki and Krebs 1996). During the last 30 years, the hypothesis that predators have a fundamental role, at least in voles cycles of northern Europe, has gained support through time‐series analyses (e.g., Hanski and Korpimäki 1995; Hanski et al. 2001), accounting models (observational data from responses and impacts, i.e., “kill” rates, of predators to vole densities) (Korpimäki and Norrdahl 1989a, 1991a; Korpimäki et al. 1991; Korpimäki and Krebs 1996) and large‐scale, long‐term experiments (review in Korpimäki et al. 2004).

Despite extensive modeling work, dedicated long‐term observational data, and large‐scale experiments that have taken huge field efforts and high costs, debate continues on whether trophic interactions, particularly whether (specialist) predators can drive small rodent cycles (e.g., Krebs 2013). Rodent populations fluctuate in many areas of the world, though not all with the same amplitude or periodicity. We argue that much of the disagreement has arisen from a misinterpretation that the low‐amplitude population cycles or seasonal fluctuations of voles in some areas of temperate Europe are analogous to the high‐amplitude and multiannual vole cycles occurring throughout boreal and arctic zones of North Europe (and also in Asia).

**FIGURE 1:**
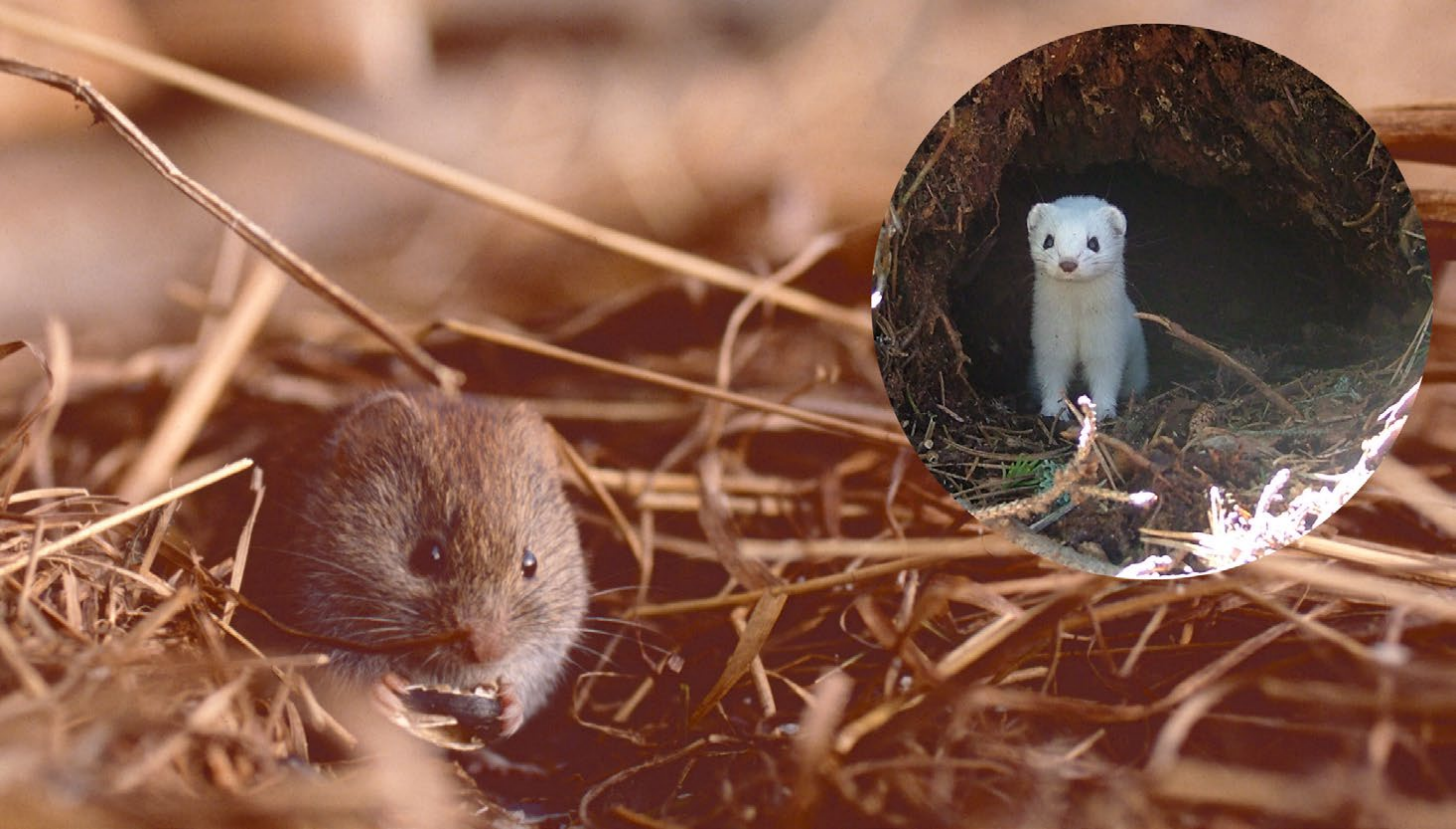
A prey and its predator: *Microtus* vole and least weasel ( *Mustela nivalis nivalis* ) in its winter coat photographed in the study area. Photo courtesy of Jalmari Lahtinen (vole) and Jorma Nurmi (least weasel).

High‐amplitude 3–5‐year vole cycles in North Europe are characterized by the following features. First, vole populations in Northern Europe usually show 50–300‐fold (sometimes even 500‐fold) differences between peak and minimum densities, whereas the corresponding difference is rarely even about 10‐fold in temperate Europe. The notably lower amplitude in temperate Europe is mainly due to higher densities during the low phase of the cycle; 40–50 voles per hectare in the south compared to sometimes almost unmeasurable low density (e.g., < 1 vole per ha) in the north. The decline to extremely low density of northern populations usually continues even in the summer, during the reproductive season of voles. Second, the spatial synchrony of vole cycles in North Europe extends 70–500 km but usually only < 20 km in temperate Europe. Third, in Northern Europe, graminivorous field voles *Microtus agrestis* and other voles of the genera *Microtus* (Figure 1; for example, the sibling vole *M. rossiaemeridionalis* and the root vole *M. oeconomys*) fluctuate in close temporal synchrony with omnivorous voles of the genus *Clethrionomys* (e.g., the bank vole *C. glareolus* and the gray‐sided vole *C. rufocanus* ) and even with insectivorous shrews, all showing their lowest densities simultaneously (Henttonen et al. 1989). In contrast, such close interspecific synchrony of small mammals has not been documented in temperate Europe (summary of characteristics of the cycles in the north and south can be found in Korpimäki, Oksanen, et al. 2005 with references). Similarly, the boom‐and‐bust dynamics of some agricultural rodent pests that are driven by climatic conditions [e.g., Australian house mouse plagues (Singleton et al. 2005) and the outbreaks of common voles *Microtus arvalis* in Central and South Europe (Jacob et al. 2020)] should be considered separately from multiannual boreal and arctic rodent cycles.

Drawing on over 40 years of research in boreal west‐central Finland—where our study site has served as a model system since the 1970s—this paper reviews observational and experimental evidence on predator–prey interactions driving high‐amplitude vole population cycles. It is not intended as a comprehensive review of the many (> 20) hypotheses proposed to explain multiannual (or seasonal and non‐cyclic) population fluctuations of small rodents, including mice, voles, and lemmings, on a global scale, which have been reviewed elsewhere. Instead, we focus on our approach to building an understanding about the role of predators in driving rodent cycles in this system. First, we report on insights gained from a long‐term data on numerical and functional responses of predators to vole population cycles and kill rates by the most significant avian and mammalian predators. These observations provided a strong foundation for a series of experiments that successfully manipulated predator abundance and vole food supply within the cyclic system. Finally, we emphasize the value of manipulative experiments in further investigating the mechanisms underlying small rodent population cycles in other regions.

## 2 Natural History of the Study System

Our long‐term study system is situated in Kauhava and Lapua, west‐central Finland (approx. 63° N, 23° E) covering about 1300 km^2^. Boreal spruce‐ and pine‐dominated forests cover 60% of the study area and 25% consist of agricultural fields (Korpimäki and Norrdahl 1989a, 1991a, 1991b). The main agricultural crops are hay, oats, and barley, and the growing season lasts from early May to early September.

Most common small mammals in the study area are bank, field, and sibling voles as well as the common shrew ( *Sorex araneus* ). Bank voles are omnivorous and common shrews insectivorous, and both of them mainly occupy forested habitats. Field and sibling voles are graminivorous (grass‐eating) and mainly occupy agricultural fields. The former prefers meadows, hay, and abandoned fields with long grass, whereas the latter thrives in cultivated fields with short grass (Norrdahl and Korpimäki 1993). Bank, field, and sibling voles all have multivoltine life histories (i.e., multiple generations per year): the most common litter size is 4–5 for bank voles and 5–6 for field and sibling voles (Norrdahl and Korpimäki 2002a).

**FIGURE 2:**
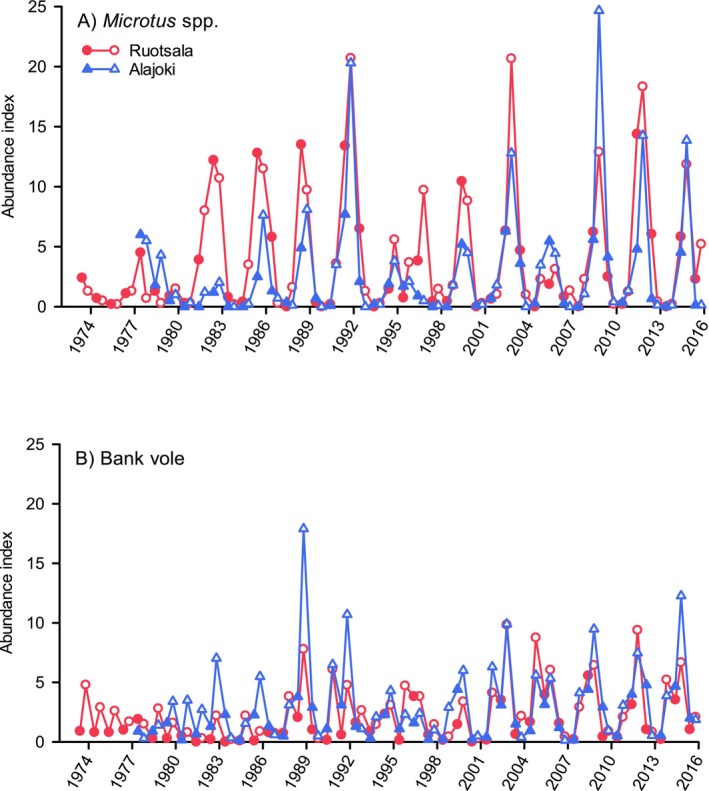
Population fluctuations of (A) *Microtus* voles (the field and sibling voles) and (B) bank voles at four sample plots (each 0.5–1.0 ha, 150–200 to 300–400 trap nights per sample plot) in both Ruotsala and Alajoki 14 km apart, west‐central Finland. Symbols denote values of spring (early May, filled symbols) and autumn (mid‐September, open symbols) trapping indices (number of individuals caught per 100 trap nights) during 1973–2015 in Ruotsala and during 1977–2015 in Alajoki (data from Korpimäki, Norrdahl, et al. 2005 and E. Korpimäki, unpublished data).

Abundance indices of voles were collected biannually (mid‐May and late September) with snap‐traps in the western (Alajoki, Lapua, during 1977–2015) and in the middle (Ruotsala, Kauhava, during 1973–2015) part of the study area (Figure 2). In these two sites, 14 km apart, four sample plots (cultivated and abandoned field as well as spruce‐ and pine‐dominated forest) were monitored (see Korpimäki, Norrdahl, et al. 2005). The snap‐trapping was conducted as a part of a regular long‐term nationwide estimation scheme of rodent densities with a standard protocol (see, e.g., Henttonen et al. 1987; Korpela et al. 2013). Permits for snap‐trapping of voles are not necessary in Finland, because they are considered pest animals (Huitu et al. 2009), and monitoring of rodents therefore complies with the guidelines from the ethics committee at the time of the study.

The increase phase of the population cycle of *Microtus* and bank voles usually occurs during spring (May) to late summer (August); the decline usually starts in early winter after the growing season of food plants and continues during the subsequent summer (Norrdahl and Korpimäki 2002a; see also Figure 2). Reproduction of *Microtus* voles can sometimes occur in winter during the increase phase of the cycle, but winter breeding has not been recorded for bank voles (Norrdahl and Korpimäki 2002a). Time‐series analyses of our monitoring data show that abundance indices of *Microtus* and bank voles as well as common shrews fluctuate in 3‐year cycles and in temporal synchrony (Korpimäki, Norrdahl, et al. 2005). This suggests a common extrinsic factor behind synchronization among population cycles of graminivorous voles, omnivorous voles, and insectivorous shrews. In addition, abundance indices of both *Microtus* voles and bank voles fluctuate in close spatial synchrony in Alajoki and Ruotsala (Figure 2). The amplitude of the population cycles of *Microtus* voles is considerably larger than that of bank voles (100–250‐fold vs. 50–100‐fold, respectively), because the peak abundance indices of *Microtus* voles are markedly higher than those of the more territorial bank voles.

Modeling the population growth of voles used this extensive dataset on age structure, sex ratio, proportion of mature individuals, litter size, and length of the breeding season in our study area (Norrdahl and Korpimäki 2002a). Importantly, cycle‐phase‐related changes in litter size had only a minor impact on the population growth rate of the three vole species. The same was true for winter breeding that occurred only in *Microtus* voles and in the increase phase. The length of the summer breeding season and the proportion of mature individuals reproducing had a positive effect on yearly population growth, but this impact was relatively weak compared to the effect of cyclic changes in survival. These results indicate that slight changes in reproductive rate are not a mechanism driving cyclic fluctuations in vole populations in North Europe (Norrdahl and Korpimäki 2002a).

Because the actual causes of vole mortality in the cycle had remained an enigma, we radio‐collared *Microtu*s and bank voles in summer in the decline and increase phases. Predators appeared to cause 99% of the summer mortality of voles, with small mustelids [least weasels *Mustela nivalis nivalis* (Figure 1) and stoats *M. erminea* ] killing 77% and avian predators 22% of radio‐collared voles (Norrdahl and Korpimäki 1995b). Short‐eared owls ( *Asio flammeus* ), Eurasian kestrels ( *Falco tinnunculus* ) and rough‐legged buzzards ( *Buteo lagopus* ) were the most important avian predators. Most notably, there were no obvious deaths from starvation or disease. The mortality by predation was higher in the decline than in the increase phase of the vole cycle (9% vs. 0.5%–2% of the vole population per day) (Norrdahl and Korpimäki 1995b). To compensate for such losses due to predation, surviving females needed to produce 0.1–1.2 offspring per female every 3 weeks in the increase phase of the vole cycle, whereas in the decline phase, the corresponding number was 11 offspring per female, which has never been observed (Norrdahl and Korpimäki 1995b). These results showed that after the stop of population growth and initial density decline in the non‐breeding season (winter), the vole‐kill rate from predators increased to a level where mortality substantially exceeded the reproductive capacity of vole prey.

## 3 Responses of Predators to Vole Cycles

Vole predators can be classified according to their functional traits as follows: resident specialists, nomadic or migratory specialists, and resident generalists (Andersson and Erlinge 1977). Of course, vertebrate predators form a continuum in both the degree of dietary specialization and mobility (Korpimäki and Krebs 1996), and these functional traits show considerable differences between arctic, boreal, and temperate regions. Nevertheless, these three categories of predators differ in their functional and numerical responses to changes in prey abundance.

**FIGURE 3:**
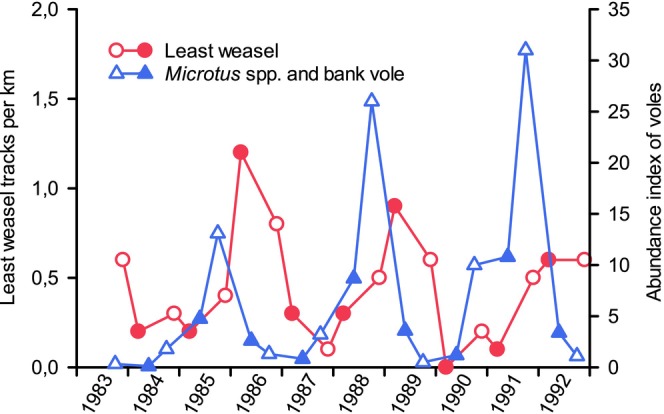
Snow‐track indices of least weasels in early (November–early December; open symbols) and late winter (late February–March; filled symbols) and pooled trapping indices (number of individuals caught per 100 trap nights) of *Microtus* and bank voles during autumn (late September; open symbols) and spring (early May; filled symbols) during 1983–1992 at Alajoki (data from Korpimäki et al. 1991 and Norrdahl 1995).

During 10 winters of snow‐tracking in our study area, the numbers of least weasels (a resident specialist) tracked the abundances of voles with a 9–12‐month time lag (Figure 3), although such lag did not occur for stoats (another resident specialist) (Korpimäki et al. 1991). However, a 9–12‐month lag has been documented for snow‐track indices of both least weasels and stoats in Northern Norway (Aunapuu and Oksanen 2003). In addition, the long‐term data set covering large areas in Finland showed that snow‐track indices of least weasels in late winter were most closely correlated with the vole abundance in the previous spring (a 9–10 month lag), while in stoats the relationship to vole abundances in the previous autumn was strongest (a 3–6 month lag) (Sundell et al. 2013). This delayed numerical response of small mustelids, particularly least weasels, is due to their limited natal dispersal, mobility, and reproduction rate (the latter when compared to that of rodents), which impacts intrinsic population growth rate. In addition, long‐distance movements of small mustelids, particularly least weasels, are risky, especially in the decline phase of the vole cycle when they often fall victims to birds of prey and red foxes ( *Vulpes vulpes* ) (Korpimäki and Norrdahl 1989b; Dell'Arte et al. 2007).

**FIGURE 4:**
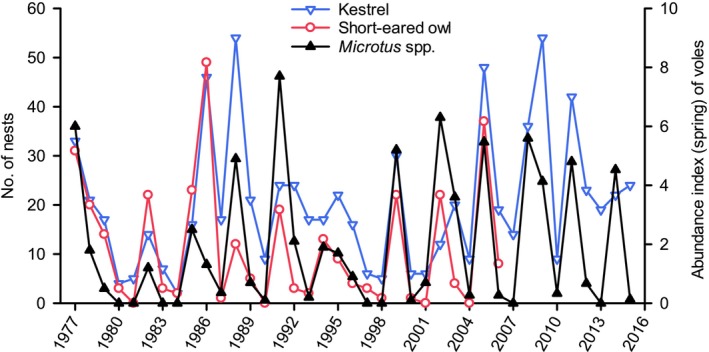
Number of nests of Eurasian kestrels during 1977–2015 and short‐eared owls during 1977–2006 at Alajoki study area (47 km^2^) as well as trapping indices (number of individuals caught per 100 trap nights) of *Microtus* voles in the spring during 1977–2015 (data from Korpimäki and Norrdahl 1991b and E. Korpimäki, unpublished data).

We also studied breeding densities of the most numerous open‐country birds of prey (migratory vole specialists) in the western part of our study area (at agricultural fields of Alajoki), generating a time series for Eurasian kestrels that extended 39 years (Figure 4), 30 years for short‐eared owls (Figure 4) and 12 years for long‐eared owls ( *Asio otus* ) (tab. 5 in Korpimäki 1992). We found that avian predator breeding densities tracked closely the abundance indices of *Microtus* voles in the spring. This is because these avian predators immediately immigrated into areas of increasing vole density but emigrated from there when vole populations started to crash. Such movements can be extensive in boreal areas. The mean natal dispersal distance of short‐eared owls is up to 1500 km (95% confidence interval 1201–1902 km) and the mean breeding dispersal distance is 470 km (255–860 km) (Calladine et al. 2012). In addition, distances of natal dispersal of both male and female offspring and breeding dispersal distances of female Eurasian kestrels can be up to hundreds of kilometers, although the mean breeding dispersal distance of males is only 6 km (Vasko et al. 2011; Valkama et al. 2014). It should be noted, however, that migratory avian predators, such as Eurasian kestrels, short‐eared owls, hen harriers ( *Circus cyaneus* ) and common buzzards ( *Buteo buteo* ) subsisting on voles as their main foods are not able to track vole densities on their breeding grounds during winter, which then results in an 8–10‐month lag (till the next breeding season) in their numerical responses to vole densities (Norrdahl and Korpimäki 2002b).

**FIGURE 5:**
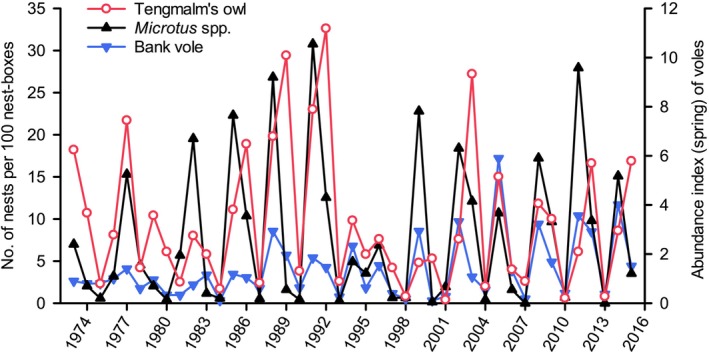
Number of nests of Tengmalm's owls per 100 nest‐boxes inspected in the Kauhava region, west‐central Finland as well as trapping indices (number of individuals caught per 100 trap nights) of *Microtus* and bank voles in the spring during 1973–2015 (data from Korpimäki and Hakkarainen 2012 and E. Korpimäki, unpublished data).

Tengmalm's owl ( *Aegolius funereus* ) is the most numerous avian predator mainly subsisting on voles in the coniferous forests of our study area. Our long‐term data of their breeding densities spans 43 years. Estimates of breeding densities tracked abundances of *Microtus* and bank voles in the spring without time lag in most years (Figure 5). This rapid numerical tracking was due to the high mobility of juveniles (maximum natal dispersal distance 600 km) and adult females (510 km), whereas adult males mostly stayed on their home ranges after their first breeding attempt (30 km) (Korpimäki and Hakkarainen 2012). In the decline phase of the vole cycle, juveniles and adult females dispersed from the area while adult males which attempted to stay on their home ranges often starved to death during the next winter (Hakkarainen et al. 2002). In this respect, Tengmalm's owl can be considered as intermediate between nomadism and residency, but in contrast to migratory specialists, Tengmalm's owls overwinter in boreal areas and are thus able to find high vole density areas also in winter. This results in notably shorter time lags in their responses to fluctuations in vole densities than in those of migratory specialists.

In contrast to specialists, resident generalist predators are able to rapidly shift to alternative prey and stay on their territories during declining densities of their main prey (Korpimäki and Sulkava 1987; Pietiäinen 1989; Korpimäki et al. 1990), which makes it unlikely that their population densities decline substantially with decreasing densities of voles. However, the proportion of breeding pairs may vary remarkably in relation to the abundance of voles; for example, only 10%–20% of Ural owl ( *Strix uralensis* ) pairs bred in years of vole scarcity in the preceding autumn, whereas 80%–90% of pairs bred in years after high vole abundance in the preceding autumn (e.g., Pietiäinen 1989). In our study area, breeding densities of resident generalist avian predators, such as Ural owls, eagle owls ( *Bubo bubo* ) and common buzzards, are relatively low. During the breeding season, these avian predators have to stay on their breeding territories from pair formation and courtship feeding through to the post‐fledging of offspring, which may result in at least a 5–6‐month time lag in their numerical responses to vole densities (Korpimäki 1994). The red fox is the most frequent generalist mammalian predator of voles in our study area (Dell'Arte et al. 2007), but we do not have long‐term data on their densities, and they are also heavily impacted by hunting mortality.

In summary, the long 9–12‐month time lags that are necessary to destabilize vole‐predator dynamics (Murdoch and Oaten 1975) have been documented for least weasels and to a lesser extent for migratory specialists, such as Eurasian kestrels and short‐eared owls. These predators are the most likely candidates to drive 3–4‐year population cycles of voles in North Europe. In addition, shorter 5–6‐month lags have been documented for nomadic specialists and resident generalists, which may then contribute to the destabilization of vole dynamics.

Differences in the functional responses of predators may also have substantial consequences for predator–prey dynamics. Some species show linear functional responses to fluctuations in the densities of voles in the spring, including breeding Tengmalm's owls, short‐eared owls, long‐eared owls, and Eurasian kestrels. These responses indicate no satiation at the highest observed vole densities and a linear density‐dependent kill rate to create an immediate predation impact on prey populations in our study area (Korpimäki and Norrdahl 1989a, 1991a; Korpimäki 1993). Linear functional responses of specialist avian predators (type I sensu Holling 1959) are in contrast with the traditional wisdom that functional responses of specialist predators are concave (type II sensu Holling 1959). In addition, two generalist avian predators (i.e., eagle and Ural owls) have concave functional responses to the fluctuating densities of voles (Korpimäki et al. 1990). This also contradicts the traditional wisdom that functional responses of generalist predators are likely to be s‐shaped (type III) with low but accelerating rates of predation at low prey densities and saturated predation rates at high densities (Holling 1959). However, it has been suggested that in fact many generalists show more of a type II functional response (Oksanen et al. 2001).

We used accounting models to show that the predation impacts of breeding Tengmalm's owls, short‐eared owls, long‐eared owls, and Eurasian kestrels were density‐dependent: larger numbers and proportions of the standing crop of *Microtus* voles were killed by these avian predators at high spring densities compared to low spring densities of voles (Korpimäki and Norrdahl 1989a, 1991a). In addition, the kill rates of *Microtus* voles by breeding short‐eared owls, long‐eared owls, and Eurasian kestrels were positively related to offspring production of voles in the course of the summer, which again indicated positive density dependence (Korpimäki and Norrdahl 1991a). Direct density‐dependent predation impact was due to a fast numerical response (newcomers dispersed to the area and produced large clutches and broods) and a type‐I functional response of breeding birds of prey (Korpimäki and Norrdahl 1989a, 1991b). Moreover, delayed density dependence was recorded for the kill rates of voles by least weasels in winter, because these kill rates were linearly related to vole densities in the previous spring (Korpimäki 1993). These results suggested that mustelid and avian predators could control the cyclic dynamics of voles. Furthermore, detailed data from numerical and functional responses, and accounting models for multiple avian and mammalian predators of non‐cyclic field voles in southern Sweden showed that a marked decrease in vole numbers from autumn to spring equaled the numbers eaten by predators during the same period, and thus the predation rate was density‐dependent in autumn (Erlinge et al. 1983, 1984).

## 4 Testing Population‐Extrinsic Factors Using Experimentation: The Predation Hypotheses

Because our observational and modeling data strongly indicated that predation mortality could be the primary density‐dependent mechanism, we conducted a series of field experiments manipulating densities of avian and mammalian predators. In addition, we also conducted experiments to find out whether food supply may be inducing direct and/or delayed density‐dependent effects on vole populations. It is important to recognize that the first of these field experiments were conducted only after the collection of extensive observational data for 15 years. This strategy would be impossible to achieve under the current short‐term regimes of research funding and policy.

First, we studied the effects of reducing numbers of the most common breeding avian predators (Eurasian kestrels and Tengmalm's owls) on *Microtus* and bank voles during 1989–1992 (Norrdahl and Korpimäki 1995a) to find out whether these predators have a limiting impact on their prey populations. We removed stick nests and nest‐boxes and filled natural cavities (i.e., potential breeding sites of birds of prey) from five reduction areas (approx. 3 km^2^ each), whereas corresponding control areas (4–15 km apart from reduction areas) had nest‐boxes in addition to natural cavities and stick nests. The yearly mean number of breeding territories of owls and kestrels was thus substantially lower in reduction than in control areas (0.2–1.0 vs. 3.0–8.2 per area). Reduction of breeding birds of prey caused only short‐term increases in density estimates of their main prey (*Microtus* voles). The densities of bank voles unexpectedly decreased in reduction areas, which we surmised to be due to increased least weasel predation pressure under the reduced breeding densities of avian predators.

**FIGURE 6:**
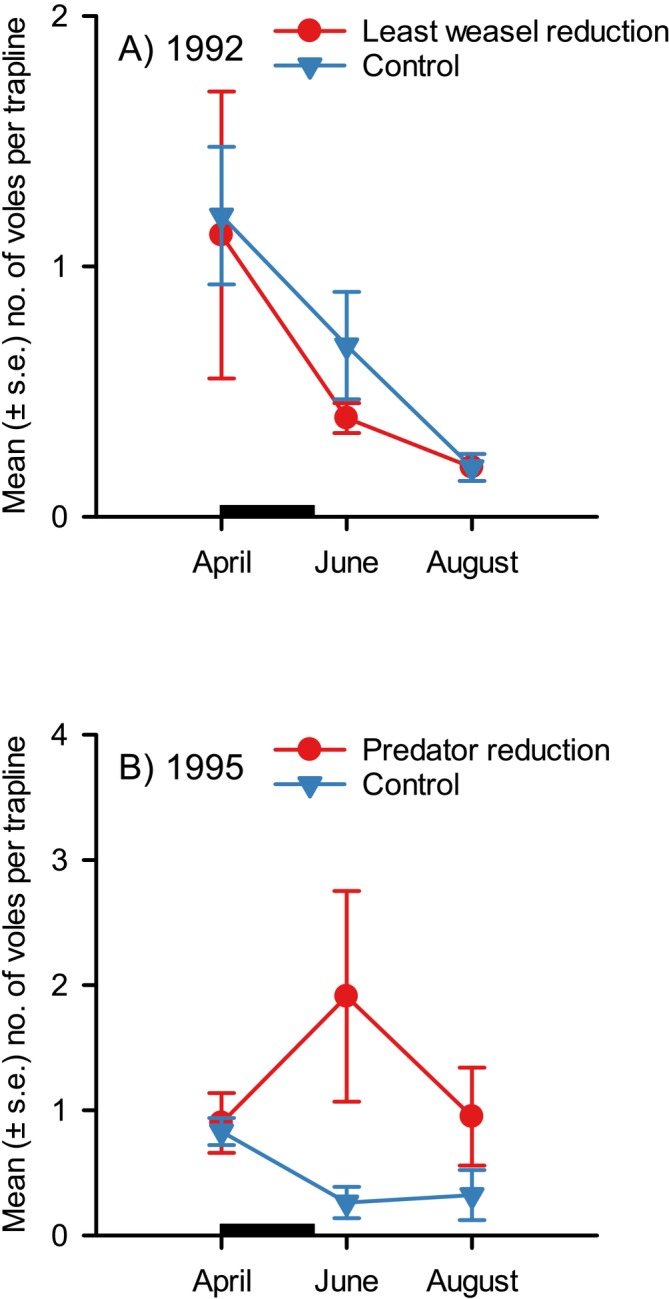
The abundance index (measured as the mean number of individuals per trapline per area) of *Microtus* voles from April to August in least weasel reduction and control areas in 1992 (A) as well as in reduction areas of small mustelids and avian predators and control areas in 1995 (B). Note that *N* = 3 (number of areas) in all cases. The black horizontal bar on the *x*‐axis refers to the period of predator reduction (data from Korpimäki and Norrdahl 1998).

Yet, why northern vole populations undergo steep crashes that begin in winter and continue during summer is still a mystery. Vole densities should not decline during their best reproductive season in summer, which is the key growth period of a wide variety of their food plants (Neby et al. 2024), unless the mortality rate is high. Predators were proposed as the main factor causing these crashes (Henttonen et al. 1987; Korpimäki et al. 1991). We tested this hypothesis by conducting another large‐scale vertebrate predator manipulation experiment, where densities of all main mammalian (the least weasel and the stoat) and avian predators (mainly the Eurasian kestrel and Tengmalm's owl) were markedly reduced in six different areas (2–3 km^2^ each), covering two crash phases (1992 and 1995) of the vole cycle (Korpimäki and Norrdahl 1998). Small mustelid numbers were reduced by live trapping, transporting, and releasing them > 30 km outside the experimental and control areas, and breeding birds of prey were reduced as in the former experiment. Significantly, the reduction of ALL main predators prevented the cyclic decline in density of voles in the subsequent summer in 1995 (Figure 6). In contrast, vole densities continued to decline where there was only least weasel reduction (in 1992) and in control areas with no predator manipulation (Figure 6). That only the reduction of all main predators was sufficient to prevent the summer crash was probably because least weasels represent < 40% of abundances of vole‐eating predators in western Finland. These results provided the first evidence for the hypothesis that synergic impacts of all main predators drive a summer decline phase of cyclic vole populations in northern Europe.

The important lesson of these two experiments was that significant density manipulation of all main mammalian and avian predators is needed to impact the population dynamics of voles. Reduction of either avian predators or least weasels likely reduced interspecific food competition among predators, which compete for preferred *Microtus* voles even in the peak phase of the population cycle (Korpimäki 1987). In addition, reduction of only avian predators probably eases their intra‐guild predation on small mustelids, particularly on least weasels in the decline phase of the vole cycle. At this time, a diverse community of predators subsisting on voles is still abundant but forced to hunt alternative prey, such as birds and least weasels (Korpimäki and Norrdahl 1989b). When birds of prey are scarce, small mustelids, particularly least weasels, might be able to compensate for mortality of voles due to birds of prey by, for example, frequent surplus killing of voles (Jedrzejewski et al. 1995).

**FIGURE 7:**
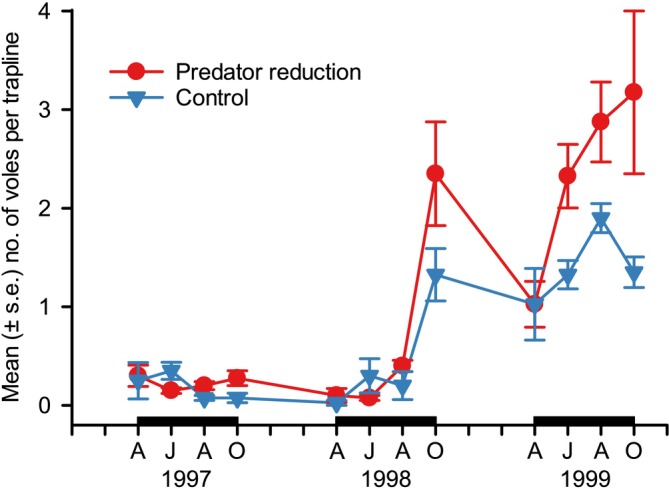
The abundance index (measured as the mean number of individuals captured per trap line per area) of *Microtus* voles from April to October (A, April; J, June; A, August; O, October) in predator reduction (solid line and filled circles, mean of four areas) and control areas (dashed line and open circles, mean of four areas) during 1997–1999. The black horizontal bar on the *x*‐axis refers to the period of predator reduction (data from Korpimäki et al. 2002).

Our third step in predator experimentation aimed to determine the synergic impacts of mammalian and avian predators in the low, increase, and peak phases of the full vole cycle during 1997–1999. We again reduced densities of all main mammalian and avian predators and compared vole abundances between four reduction and four control areas (each 2.5–3 km^2^). A significant reduction of predators from early spring (April) to late autumn (October) was conducted yearly using the same methods as in the second experiment. The reduction of predator densities increased the autumn density of *Microtus* voles fourfold in the low phase, accelerated the increase phase twofold, increased the autumn density of voles twofold in the peak phase, and delayed the initiation of decline of the vole cycle (Figure 7; see also Korpimäki et al. 2002). Notably, the decline of *Microtus* voles in the peak phase of the population cycle apparently began from August onwards in the control areas, whereas in the reduction areas vole abundances continued to increase from August to October. Predator reduction also increased densities of bank voles in the low and increase phases of the vole cycle but not in the peak phase, which indicated that predators successively reduce the densities of both main prey (*Microtus* voles) and alternative prey species (bank voles) after the peak phase of vole population cycles, thus inducing a synchronous low phase (Korpimäki, Norrdahl, et al. 2005). Based on this experiment, we ran demographic models that predicted vole dynamics would change from regular multiannual cycles to annual fluctuations with a declining impact of the delayed density‐dependency imposed by vole‐eating predators (Korpimäki et al. 2002).

An alternative but uncommon way to understand predation impact is to add predators to a system, which has been used in other studies of predation and vole cycles. A large‐scale introduction experiment was conducted during 1997–2000 in south‐eastern Finland where least weasels were released on three large ‘predator addition’ islands (6–9 km^2^ each) and three comparable adjacent islands served as controls (Sundell 2003). The novel idea behind this study was to eliminate the time lag in the numerical response of least weasels by adding weasels during the early increase phase of the vole cycle. Although a total of 159 weasels were released, the shortcoming of the experiment was that captive‐born least weasels soon starved to death or were killed by larger predators in nature. As a result, there was only an obvious treatment effect on population dynamics of voles on one of the three treatment‐control island pairs (Sundell 2003). The weak efficacy of the least weasel‐density manipulation in this island experiment in Finland thus resembles the common weasel ( *Mustela nivalis vulgaris* ) reduction experiment in Kielder Forest, U.K. (Graham and Lambin 2002) that failed to substantially alter predator densities because of re‐invasion of weasels into small reduction areas (Korpimäki, Oksanen, et al. 2005). The results of both these experiments highlight the importance of effective predator density manipulation when experimentally studying the impact of predation. Indeed, the worldwide meta‐analysis of predation manipulation experiments found that the efficiency of predator density treatment was a main determinant of its effect size (Salo et al. 2010).

## 5 Predation and Spatial Synchrony of Vole Population Cycles

Our first experiment reducing avian predator numbers shed new light on an interesting puzzle of northern vole cycles, namely spatial synchrony of their fluctuations at a scale of hundreds of square kilometers (e.g., Elton 1942; Kalela 1962). The following three, definitely not mutually exclusive, explanations for this synchrony are (1) climatic factors similarly affecting reproduction and survival of small mammals at a large scale (Moran Effect after Moran 1953; see also Sinclair et al. 1993), (2) nomadic and migratory predators concentrating on high‐density prey patches, and thereby reducing the prey density of these patches close to the average density of a larger area (Ydenberg 1987; Korpimäki and Norrdahl 1989a). The spatial synchrony may also arise (3) as a result of dispersal of voles between populations (e.g., Bjørnstad et al. 1999).

In our experiments, hunting birds of prey indeed concentrated in high‐density prey areas after their breeding season (August to September), but not necessarily during the breeding season (April to June), when they were constrained to hunt in the vicinity of their nests. The experimental reduction of breeding avian predators increased variation in prey density between the five reduction and the five control areas but not within areas of the same treatment level (Norrdahl and Korpimäki 1996). The difference in variation between avian predator reduction and control areas was largest in the late breeding season of birds of prey and decreased rapidly after the breeding season due to the influx of migrating diurnal raptors and owls in these areas. These results thus appeared to support the hypothesis that the spatial synchrony of population cycles in small mammals may be driven by nomadic and migratory avian predators concentrating in high prey density areas (Norrdahl and Korpimäki 1996).

We further conducted a field experiment to evaluate whether climate‐related environmental effects, avian and mammalian predation, and dispersal were important mechanisms in inducing spatial synchrony among cyclic populations of field voles (Huitu et al. 2005). The study was conducted during the increase and peak phases on four agricultural field sites situated 1.5–7.0 km apart. Each field contained two 0.5‐ha fenced enclosures and one 1‐ha unfenced control area. One enclosure per field allowed access by small mustelid predators and the other by avian predators; all enclosures prevented the dispersal of voles. The unfenced control areas allowed access by all predators as well as dispersal by voles. Treatment‐wise asynchronous vole populations were created by transplanting different numbers of voles to different enclosures before the predator access treatments were applied. The growth rates of all enclosed populations were tightly synchronized during the course of the experiment. Conversely, synchrony both among the unfenced populations and between the fenced and unfenced populations was practically non‐existent. During winter, in the increase phase of the cycle, all experimental vole populations declined to low densities due to a seasonal effect of winter food depletion (Huitu et al. 2005). During summer of the peak year, all experimental populations fluctuated in synchrony and both small mustelids and birds of prey appeared to be abundant enough to induce synchrony. Dispersal was identified as a potential contributor to synchronization, but the magnitude of its effects could not be reliably discerned. Our results indicate that spatial synchrony among cyclic northern vole populations may be induced by predation and seasonality (food depletion in winter), acting successively during different seasons and phases of the vole cycle. In addition to mobile avian predators, small mustelids were able to synchronize population dynamics of field voles at the scale of < 10 km (Huitu et al. 2005). However, migratory and nomadic avian predators are more rapidly able to find dense patches of vole populations in comparison to terrestrial predators, and are thus the main synchronizing agents of population fluctuations of voles, especially at the larger scale.

The two above‐mentioned experimental results appeared to support the hypothesis that the spatial synchrony of population cycles in voles is driven by nomadic and migratory avian predators concentrating in high prey density areas, and thereby reducing the prey density of these patches close to the average density of a larger area (Ydenberg 1987; Korpimäki and Norrdahl 1989a). Further support for this hypothesis was received in an elegant study where Ims and Andreassen (2000) distinguished between local dispersal and regional predation mechanisms of spatial synchrony of experimental vole populations in Norway. The fates of 481 radio‐tagged voles showed that predation by mobile birds of prey (long‐eared and short‐eared owls, Eurasian kestrels and rough‐legged buzzards) was the main mortality factor, highest in the crash phase for all populations, and thus the synchronizing mechanism (Ims and Andreassen 2000). One biological mechanism behind the Moran effect in seasonal environments may be food depletion in winter, whereas dispersal of voles is probably the least important mechanism inducing spatial synchrony of vole cycles in extensive areas.

## 6 Testing Population‐Extrinsic Factors: Food Hypotheses

Experiments conducted with snowshoe hares ( *Lepus americanus* ) showed that the interactive effects of predation and food shortage were driving their 10‐year population cycles (Krebs et al. 1995). Delayed density‐dependent effects of food shortage have also been proposed as factors driving population cycles of voles, along with predation mortality (e.g., Agrell et al. 1995). However, long‐term food supplementation on one large grid and the non‐supplemented control grid was unable to prevent the steep decline of bank voles in Finnish Lapland (Yoccoz et al. 2001).

**FIGURE 8:**
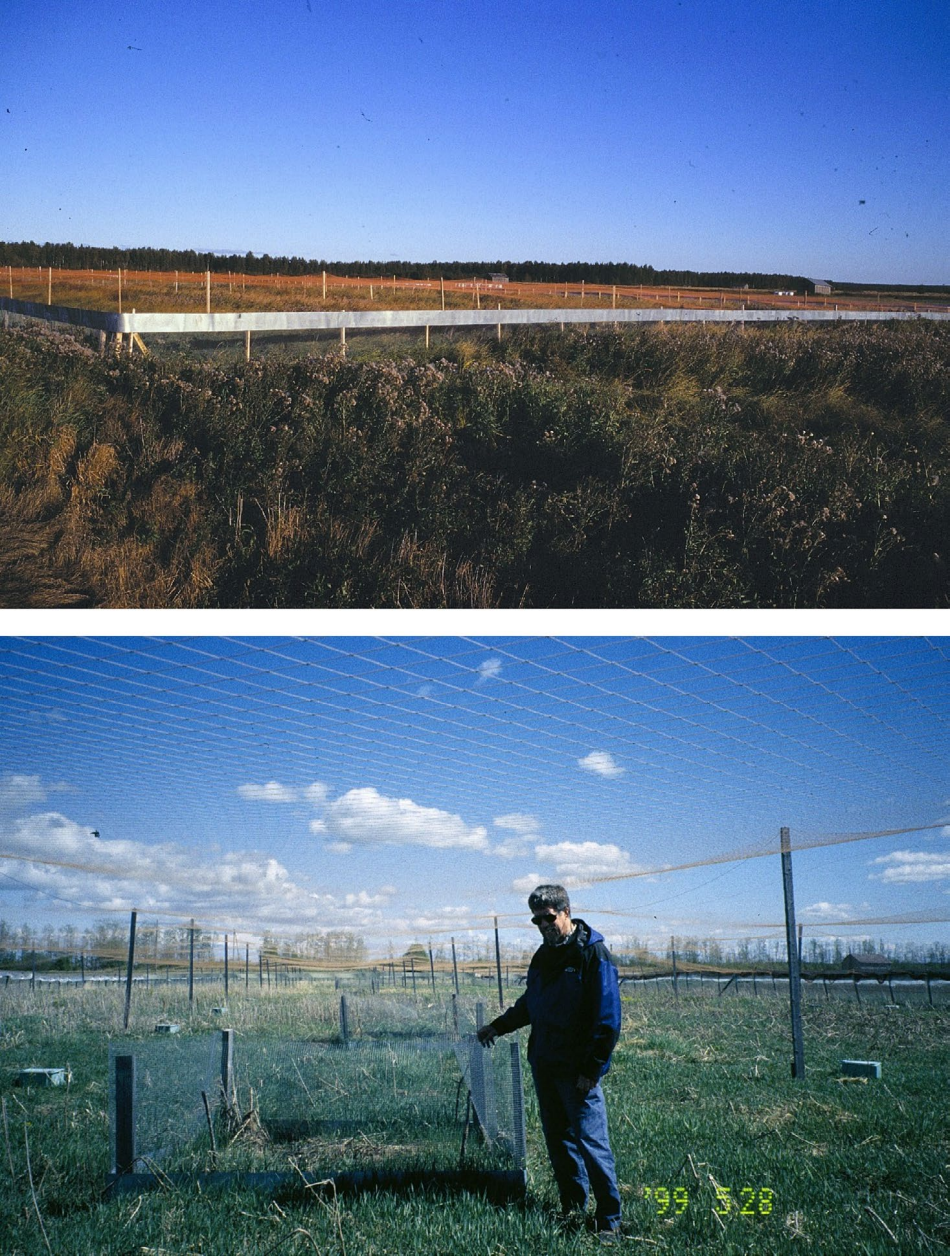
In spring 1996, four 1‐ha enclosures were constructed in agricultural fields of Alajoki, western Finland (Klemola, Koivula, et al. 2000). Adjacent unfenced 1‐ha areas served as controls where predators had free access. The pairs of enclosures and their control areas were within 12 km^2^ and at least 1.5 km apart. The enclosures were constructed using hard‐ware cloth (12.7 mm mesh), which extended 0.5 m below ground and 1.3 m above ground. A 40‐cm wide metal sheet on the upper edge of the fence was fastened to prevent climbing by mammalian predators. To prevent access of avian predators, the enclosures were covered with nylon net (10‐cm mesh; upper panel). Quantity of winter food of voles was assessed by counting green shoots of wintering grasses and dicotyledons from permanent sampling plots (2 m × 2 m) in the enclosures and control areas during the experiment. Half of the plots were fenced to protect them against grazing by voles. Six protected and six unprotected plots were located in each enclosure, and five protected and five unprotected plots in each control area. Professor Charles Krebs at the plot protected against grazing inside the fence on May 28, 1999 (lower panel).

Our year‐round predator‐proof fences and their 1‐ha unfenced control areas (as in Huitu et al. 2005) were used to study the interactive effects of predation and food quantity on two *Microtus* voles during 1996–1998 (Figure 8, see also Klemola, Koivula, et al. 2000). Here, predator exclusion induced rapid population growth of voles in autumn 1996 and increased the peak abundance of voles over 20‐fold in comparison to unfenced control areas in late summer 1997, but the enclosed populations crashed close to zero due to food shortage during the winter 1997–1998. After both winters, the number of green shoots of plants was significantly lower in the predator‐proof fences than in the control areas with free access to predators, suggesting grazing in the fences reduced food availability. In addition, the number of green shoots in the fences was also lower in unprotected than grazing‐protected plots, but this difference did not occur in the unfenced control areas. Shoots in unprotected plots in the fences were also more damaged (ca. 90% of shoots were clipped by voles) than shoots in the controls or in protected plots (< 10%) (Klemola, Koivula, et al. 2000). In addition, heavy winter grazing by voles also reduced the biomass of available vegetation and killed woody species (willows), which implies that voles were suffering from serious food shortage (Norrdahl et al. 2002). Despite the high vole densities in summer and autumn 1997, the proportion of reproducing individuals of all potentially reproductive females remained high in the fenced populations throughout the summer and autumn (Klemola, Koivula, et al. 2000). Thus, this fence experiment did not support either deteriorated food quality or quantitative insufficiency of summer forage as delayed density‐dependent factors in the generation of the vole cycles (Klemola, Norrdahl, et al. 2000).

We did not find detrimental effects of previous heavy wintertime grazing on population growth, reproduction, or body condition of field voles in the following summer. Furthermore, chemical analyses did not show consistent effects of grazing on main nutritional components (protein, sugar, fiber) of common food plants (grasses) (Klemola, Norrdahl, et al. 2000). These results suggested that population cycles of *Microtus* voles in grassland habitats are not primarily driven by delayed effects of the plant‐herbivore interactions. The rapid regrowth of graminoids (Turchin and Batzli 2001), even after high vole densities and heavy grazing in winter, does not align with the long time lags that may generate the 3–5‐year vole cycles at northern latitudes (Klemola, Koivula, et al. 2000; Klemola, Norrdahl, et al. 2000).

## 7 Experiments on Interactive Effects of Predators and Food Supply

**FIGURE 9:**
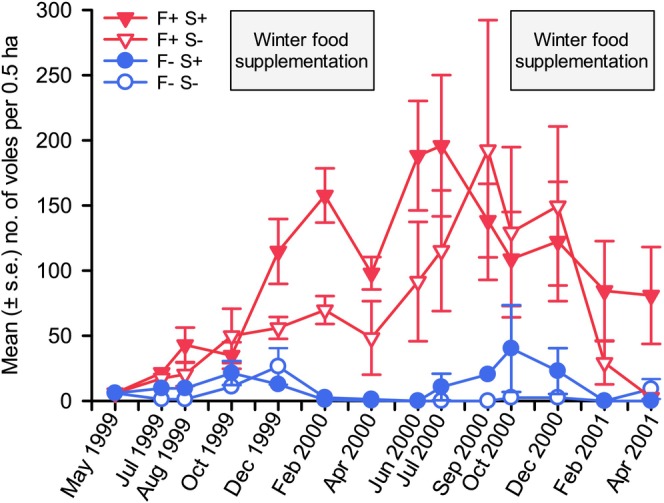
Estimates of vole abundance in fenced (predator exclusion) and unfenced (control, predators present) areas with and without winter food supplementation. F+, with fencing; F−, no fencing; S+, winter food supplementation, S−, no winter food supplementation. Sample sizes are *n* = 4 areas each for F + S+ and F + S− treatments, and *n* = 2 areas each for F−S+ and F−S− treatments (data from Huitu et al. 2003).

Since our experimental results (summarized above) suggested predation as the most likely mechanism affecting vole populations in a delayed density‐dependent manner and food supply in the direct density‐dependent manner, our team tested this hypothesis by conducting a two‐factor experiment in our predator‐proof fences and comparable control areas in 1999–2001. This time both predation rate and winter food supply were manipulated. Field vole populations in fenced predator exclosures rapidly attained higher densities than in unfenced areas, with the difference persisting until the end of the experiment (Figure 9). In the first winter, food supplementation increased vole population growth in fenced areas, but not in unfenced areas (Huitu et al. 2003). The growth of vole populations in both supplemented and non‐supplemented fenced areas became limited in a direct density‐dependent manner during the first winter. During the second winter, food supplementation prevented the crash of vole populations within fences, whereas again no obvious effect was found in the areas exposed to predation. Furthermore, supplemental winter food increased the overwinter survival of voles in fenced areas in both winters (Huitu et al. 2003). These results showed that field vole populations that have escaped regulation by predators are limited in growth by a shortage of winter food. Winter food shortage may thus be a candidate for the direct density dependence inherently necessary for the occurrence of population cycles. Results of our experimental tests of food hypotheses are consistent with those from the High Arctic food web in Svalbard: in the absence of top‐down regulation (i.e., without predators) *Microtus* vole populations exhibited high‐amplitude, non‐cyclic fluctuations, partly driven by weather stochasticity (Fauteux et al. 2021).

## 8 Testing Population‐Intrinsic Hypotheses

Intrinsic hypotheses to explain cyclic fluctuations of small mammal populations propose that population growth is self‐regulated within the population (e.g., Chitty 1960). Boonstra et al. (1998) suggested that the senescence‐maternal effects hypothesis remains the most plausible of intrinsic explanations for population cycles in voles. This hypothesis proposes that a detrimental change (e.g., a shift in the age structure of reproductive females) in maternal quality occurs in the peak phase, carries over several generations, and leads to population decline and a subsequent low phase of the population cycle (Boonstra et al. 1998).

In early spring 1998, field vole populations were in the synchronous low phase in large areas in western‐central Finland (Klemola, Koivula, et al. 2000). According to intrinsic hypotheses, low‐phase voles should reproduce poorly (Boonstra et al. 1998). We live‐trapped and introduced field voles to four enclosures and corresponding control areas in May 1998. Although graminoids in these enclosures had suffered heavy grazing in the preceding winter, introduced low‐phase voles increased to higher densities than comparable voles in previously ungrazed control areas that were exposed to predators (Klemola, Norrdahl, et al. 2000; Klemola et al. 2002). These results showed that even if these voles had a slower reproduction rate, it did not noticeably affect the overall dynamics, and when they were protected from predation, they bred well. These results were consistent with the results of elegant transplantation experiments by Ergon et al. (2001) which also showed that life‐history traits of field voles in a fluctuating population rather respond to the immediate environmental conditions than show memory of past conditions.

## 9 Limitations of Our Experiments

All ecological field experiments are to various extents compromises of temporal and spatial scales, replication, and funding. Of these, spatial and temporal scales have been the most important to our team: experiments should be performed at spatial and temporal scales relevant to the processes under study. Replication can also be attained afterwards by conducting a similar experiment elsewhere (Oksanen 2001), but if the manipulation is in too small a spatial and temporal scale, it is non‐successful and cannot be mended afterwards.

In our predator reduction experiments in large unfenced areas, we could not reduce small mustelids and avian predators during winter due to logistic reasons. Kill trapping of mustelids was not allowed, and wintertime live‐trapping is quite impossible because of trap mortality due to coldness. In addition, efficient live‐trapping of avian predators is practically impossible, and hunting is naturally illegal throughout the whole year. Large spatial scale and financial and logistic constraints also prevented us from estimating survival rates of voles by live‐trapping in unfenced predator reduction and control areas, but vole densities that are the product of survival and reproduction rates could be estimated by snap‐trapping.

## 10 Conclusions and Future Prospects

On the basis of our unique series of large‐scale, replicated field experiments in both unfenced and fenced settings, where densities of vole predators were significantly reduced, we conclude that the collective impact of both mustelid and avian predators is a probable mechanistic explanation for high‐amplitude population cycles of voles in boreal regions of North Europe. In these seasonal environments, where the growing season is short, the shortage of high‐quality winter food may be the directly density‐dependent factor stopping the reproduction and growth of vole populations, possibly acting simultaneously with other limiting factors (e.g., winter predation by small mustelids and other mammalian predators as well as nomadic and resident avian predators, competition, stress, and social factors which may be vole‐species‐specific) as discussed earlier (e.g., Andreassen et al. 2021). This allows vole predators to catch up with prey densities and impose population decline and prolong the low phase of the cycle in a delayed density‐dependent manner. To be clear, limiting, directly density‐dependent factors, whatever they are, do not cause the cyclic vole dynamics per se, but delayed effects are needed.

Considering the importance of predators, our results are consistent with the results of three large‐scale experimental studies on predator reduction in Canadian arctic tundra (Reid et al. 1995; Wilson et al. 1999; Fauteux et al. 2016), as well as two long‐term mammalian predator reduction experiments in the tundra of Northern Norway (Ekerholm et al. 2004; Hambäck et al. 2004). These experimental studies supported predation as a driving mechanism in lemming cycles in Canadian tundra and in high‐amplitude vole cycles in Northern Norway, whereas the food quantity hypothesis did not receive much support in Canada (Gauthier et al. 2025) but received some support in Norway (Hambäck et al. 2004). However, there is an urgent need to conduct additional two‐factor experiments on a large spatial scale to find out the interactive or additive effects of predator reduction and food supplementation on cyclic population fluctuations of voles.

Our experimental results, together with those by Ims and Andreassen (2000), support the hypothesis that the large‐scale geographic synchrony of vole cycles is likely driven by nomadic and migratory avian predators concentrating in high prey density areas, thereby reducing the prey density of these patches close to the average density of a larger area. In addition, we found no support for intrinsic factor hypotheses as explanations of high‐amplitude vole cycles because voles from the low phase reproduced immediately and rapidly increased in numbers when protected from predation. Although some evidence for food limitation in winter was observed, we do not suggest food‐plant related hypotheses as principal explanations of northern vole cycles because no lagged effects of overgrazing on population growth of voles were found in our experiments. Generally, more observational and experimental research on the winter ecology of voles, small mustelids, and other predators is needed, although being challenged by long, cold, and snowy winters in boreal and arctic regions. Subnivean camera traps may be a useful new tool to further study the interactions of voles and their small mustelid predators in winter (e.g., Kleiven et al. 2023). Remote tracking of wide‐ranging avian predators, such as short‐eared owls, snowy owls ( *Bubo scandiacus* ) and Eurasian kestrels could also help reveal how these nomadic or migratory avian predators rapidly find high vole density areas and may thus be able to induce geographic synchrony of vole populations (e.g., Therrien et al. 2014; Calladine et al. 2024).

Small rodents show population cycles in many other parts of the world with different predator and prey communities and different patterns of plant growth. We encourage researchers to use manipulative experiments (i.e., mechanistic approach, sensu Krebs 1995) to further study mechanisms generating vole cycles elsewhere, for example, in the boreal–arctic zones of Eurasia and in temperate Europe. In temperate Europe, vole cycles have low amplitude, relatively higher density in the low phase, and restricted interspecific and spatial synchrony. There, the predator guild is relatively species‐rich, and both mammalian and avian predators, particularly resident generalists, are more abundant than in North Europe. The absence of deep snow cover also allows heavy impacts of multiple predators on voles in winter. To test the synergic predation hypothesis in temperate Europe and elsewhere, we challenge colleagues to conduct the critical experiment where the collective density of all main avian and mammalian predators is effectively manipulated at the proper spatial and temporal scale with sufficient replication. Nowadays, this should be achieved by non‐lethal means, for example, by enclosing the sites with a fence and roof. This method excludes large predators but allows the movement of voles and small mustelids, which can be manipulated by removal trapping, covering sufficiently large areas around the fenced grids. When all main predators are significantly reduced, the synergic predation hypothesis predicts that the amplitude of the cycle will substantially decline in reduction sites compared to control sites, because low densities of voles will increase and peak densities, in turn, will decline in reduction areas. One important lesson from our predator reduction experiments is that single‐species predator manipulations may turn out to be competition experiments among predators: the predicted response then is that other predators increase their use of the treatment areas and prevent any detectable impacts on vole dynamics.

After 100 years of research, the heart of high‐amplitude vole cycles is still beating in our study area and elsewhere in Finland (Figure 2; see also Korpela et al. 2013; fig. 1 in Andreassen et al. 2021), although in some areas of the boreal and arctic zones of Eurasia, vole cycles may be disappearing (e.g., Hörnfeldt et al. 2005). The future prospects of high‐amplitude vole cycles in Northern Europe are obscure, especially under ongoing climate change. Several species of once numerous vole‐eating predators are also declining in boreal and arctic areas. Populations of stoats, least weasels, and Tengmalm's owls have declined by 60%–70% in Finland from the 1980s (Korpimäki and Hakkarainen 2012; Sundell et al. 2013), and populations of three weasel species have also drastically declined in North America (Jachowski et al. 2021). In addition, severe decreases of short‐eared owl and snowy owl populations have been recorded in both Europe and North America (Fernández‐Bellon et al. 2020; Miller et al. 2021; Gousy‐Leblanc et al. 2023; McCabe et al. 2024), hen harrier populations have declined in Europe (Fernández‐Bellon et al. 2020), and common buzzards, rough‐legged buzzards, and Eurasian pygmy owls ( *Glaucidium passerinum* ) have declined at least in Northern Europe (Lehikoinen et al. 2009; Terraube et al. 2014; Korpimäki et al. 2020). The apparent main reasons for the considerable population decline of these predators are habitat degradation and loss, as well as climate change and/or interactive effects of these factors. Under a scenario of decreasing predator populations and a prolonged growing season inducing reduced food limitation on voles, our experimental results predict that the period of vole cycles may shorten, which may result in only seasonal fluctuations of vole populations. We have been privileged to study voles, their multiannual cyclic population fluctuations, and the diverse assemblage of predators for decades, and hope that this overview provides a framework for future research projects.

## Author Contributions

**Erkki Korpimäki:** conceptualization (lead), data curation (lead), formal analysis (equal), funding acquisition (lead), investigation (lead), methodology (lead), project administration (lead), writing – original draft (lead), writing – review and editing (lead). **Peter B. Banks:** conceptualization (equal), data curation (supporting), formal analysis (supporting), funding acquisition (supporting), investigation (supporting), methodology (supporting), project administration (supporting), writing – original draft (supporting), writing – review and editing (equal). **Tero Klemola:** conceptualization (equal), data curation (equal), formal analysis (equal), funding acquisition (supporting), investigation (supporting), methodology (supporting), project administration (supporting), writing – original draft (equal), writing – review and editing (equal).

## Conflicts of Interest

The authors declare no conflicts of interest.

## Supporting information


Appendix S1

## Data Availability

If accepted for publication, the data used for Figures 2, 3, 4, 5, 6, 7 and Figure 9 will be archived to *figshare* data repository, https://figshare.com. All the required data are uploaded as Supporting Information.
