# Supplementary material for: Insights From Observations and Large‐Scale Field Experiments on Vole Population Cycles in Northern Europe: A 40‐Year Study of Predator–Prey Interactions
Source: Ecol Evol. 2025 May 8;15(5):e71419. doi: 10.1002/ece3.71419 (PMC12059622; doi:10.1002/ece3.71419)
Supplement: Supplementary file 1 — Appendix S1 [file ECE3-15-e71419-s001.docx]

Appendix S1
